# Nuclear Galectin-1 Drives Cancer Progression through *O*-GlcNAcylation-Dependent Regulation of SOX2

**DOI:** 10.7150/ijbs.124928

**Published:** 2026-04-16

**Authors:** Woong Kim, Ye-Seal Yim, Jung-Hwan Baek, Young Soo Park, Ji-Joon Song, Joon-Yong Chung, Jungsoo Gim, Seok-Jun Kim, Kyung-Hee Chun

**Affiliations:** 1 Institute of Well-Aging Medicare & Chosun University G-LAMP Project group, Chosun University, Gwangju 61452, Republic of Korea.; 2 Department of Biochemistry & Molecular Biology, Graduate School of Medical Science, Brain Korea 21 Project, Yonsei University College of Medicine, 50-1 Yonsei-ro, Seodaemun-gu, Seoul 03722, Republic of Korea.; 3 JLBiotherapeutics Inc., Magokjungang-ro 168, Kangseogu, Seoul 07789, Republic of Korea.; 4 Department of Pathology, Asan Medical Center, University of Ulsan College of Medicine, Seoul 05505, Republic of Korea.; 5 Department of Biological Sciences, KI for BioCentury, Korea Advanced Institute of Science and Technology (KAIST), 291 Daehakro, Yuseong-gu, Daejeon 34141, Republic of Korea.; 6 Molecular Imaging Branch, Center for Cancer Research, National Cancer Institute, National Institutes of Health, Bethesda, MD 20892, USA.; 7 Department of Biomedical Science, Chosun University, Gwangju 61452, Republic of Korea.; 8 Department of Integrative Biological Sciences & BK21 FOUR Educational Research Group for Age-associated Disorder Control Technology, Chosun University, Gwangju 61452, Republic of Korea.; 9 Affiliate faculty, Pohang University of Science and Technology, Pohang 37673, Republic of Korea.

**Keywords:** Galectin-1, SOX2, *O*-GlcNAcylation, cancer stemness

## Abstract

Galectin-1 is frequently upregulated in tumors and contributes to cancer progression. Here, we identify galectin-1 as a critical regulator of cancer stem-like properties. Silencing galectin-1 suppressed proliferation, motility, side population fraction, and tumorsphere formation *in vitro*, and impaired tumor initiation and growth *in vivo*, whereas overexpression enhanced these malignant phenotypes. Transcriptomic profiling revealed stemness-associated transcription factors as major downstream targets, with SOX2 emerging as a key effector. Galectin-1 knockdown reduced SOX2 expression, whereas overexpression increased SOX2 nuclear abundance and transcriptional activity. Rescue experiments demonstrated that SOX2 is functionally required for galectin-1-mediated stemness and tumorigenesis. Mechanistically, galectin-1 associates with SOX2 in an *O*-GlcNAcylation-dependent manner. Inhibition of *O*-GlcNAcylation or mutation of SOX2 *O*-GlcNAc sites disrupted this interaction, reduced SOX2 transcriptional activity, and impaired tumorsphere formation, supporting an intracellular lectin-like function. Structural modeling predicted that residues E71 and R73 within the carbohydrate recognition domain are critical for carbohydrate-mediated recognition of *O*-GlcNAc-modified SOX2, which was validated by mutagenesis. Clinically, galectin-1 was highly expressed in gastric tumors, correlated with advanced stage, and predicted poor prognosis. Notably, high co-expression of galectin-1 and SOX2 was significantly associated with unfavorable survival outcomes. These findings establish galectin-1 as a reader-like protein that functionally engages *O*-GlcNAcylated SOX2 and highlight the galectin-1/SOX2 axis as a potential therapeutic target in gastric cancer.

## Introduction

Galectins are a family of carbohydrate-binding proteins known as S-type lectins, characterized by high affinity for β-galactosides and the ability to mediate cell-cell and cell-matrix interactions through binding to cell-surface glycoproteins 1. Fifteen galectins have been identified, and aberrant expression of several family members has been linked to cancer development, progression, and metastasis 2, 3. Among them, galectin-1 contains a single carbohydrate recognition domain (CRD) and can function as either a monomer or homodimer 4. Elevated galectin-1 expression has been associated with key oncogenic processes, including cell aggregation, metastatic dissemination, angiogenesis, and resistance to apoptosis 5-7. Moreover, galectin-1 has been proposed as an independent prognostic factor for cancer-specific survival 8.

Although galectin-1 has been primarily characterized by its extracellular glycan-binding functions, accumulating evidence indicates important intracellular roles in multiple malignancies. Galectin-1 promotes invasion and epithelial-mesenchymal transition through non-canonical Hedgehog signaling 9, enhances stem-like properties in an H-Ras-dependent manner 10, and activates integrin-FAK and PI3K-AKT-mTOR pathways to drive invasion, drug resistance, and stemness maintenance 11, 12. It can also augment WNT/β-catenin signaling to sustain cancer stemness and therapy resistance. Notably, nuclear galectin-1 has been reported to regulate oncogenic transcriptional programs through interaction with chromatin modifiers such as MLL1 13. In addition, galectin-1 contributes to immune regulation by inducing nuclear translocation of endonuclease G and modulating immune-tolerance-related gene expression 14, 15. Collectively, these findings suggest that galectin-1 is not restricted to extracellular glycan recognition but can participate in intracellular signaling and transcriptional regulation.

In our study, galectin-1 knockdown markedly reduced proliferation, invasion, migration, and the side population fraction in gastric cancer cells. Transcriptomic profiling revealed that the most significant transcriptional alterations following galectin-1 depletion involved stemness-associated transcription factors, suggesting a potential link between galectin-1 and cancer stem-like programs. Cancer stem cells (CSCs), defined by their capacity for self-renewal, differentiation, and resistance to conventional therapies, are recognized as key drivers of tumor progression and therapeutic failure 16. Core transcription factors such as SOX2, OCT4, NANOG, KLF4, and c-MYC orchestrate CSC programs by maintaining pluripotency and stem-like phenotypes, and their dysregulation is strongly associated with aggressive disease behavior and poor prognosis across multiple malignancies, including gastric cancer 17, 18.

Despite extensive studies on galectin-1 in tumor biology, whether and how galectin-1 functionally engages CSC-related transcription factors remains incompletely understood. Previous investigations have largely focused on extracellular signaling, EMT induction, immune modulation, or metabolic reprogramming, whereas a direct intracellular mechanism linking galectin-1 to stemness-associated transcriptional regulation has not been fully elucidated. Based on our findings, we hypothesized that nuclear galectin-1 functionally interacts with and modulates the activity of stemness-associated transcription factors in an *O*-GlcNAc-dependent manner, thereby sustaining CSC traits and promoting gastric cancer progression.

## Materials and methods

### Cell culture

Eleven human gastric cancer cell lines (AGS, MKN28, YCC-2, KATOIII, SNU1, SNU16, SNU216, SNU601, SNU638, SNU668, and SNU719) were obtained from the Korea Cell Line Bank and cultured in RPMI 1640 medium supplemented with 5% fetal bovine serum (Corning Cellgro, Manassas, VA, USA) and 1% antibiotics (Gibco-BRL, NY, USA), as described previously 19. Cells were maintained at 37 °C in a humidified incubator with 5% CO₂.

### Cell proliferation assay

Cells were seeded in 96-well plates at 3 × 10³ cells per well. After 24 h, cells were transfected with control siRNA (scRNA), galectin-1 siRNA, empty vector, or galectin-1 expression vector. Forty-eight hours after transfection, cell viability was assessed using the WST-based EZ-Cytox assay kit (DoGenBio, Seoul, Korea) according to the manufacturer's instructions.

### Gene knockdown and overexpression

Galectin-1 and SOX2 knockdown was performed using siRNA or lentiviral shRNA systems. Galectin-1 and SOX2 siRNAs were synthesized by GenePharma (Shanghai, China). Lentiviral shRNA constructs (MISSION® shRNA) were obtained from Sigma-Aldrich. The sequences were as follows: Galectin-1 siRNA #1: 5′-CCAACACCAUCGUGUGCAA-3′, Galectin-1 siRNA #3: 5′-AGGCCAACCUGACCGUCAA-3′, Galectin-1 shRNA #3: 5′-CCGGACGGTGACTTCAAGATCAAATCTCGAGATTTGATCTTGAAGTCACCGTTTTTTTG-3′, Galectin-1 shRNA #4: 5′-CCGGGTGTTGCAGAGGTGTGCATCACTCGAGTGATGCACACCTCTGCAACACTTTTTTG-3′, SOX2 shRNA: 5′-CCGGACGGTGACTTCAAGATCAAATCTCGAGATTTGATCTTGAAGTCACCGTTTTTTTG-3′. Cells were transfected with 20 nM siRNA using Lipofectamine RNAiMAX (Invitrogen, Carlsbad, CA, USA) and harvested 48 h post-transfection as described previously 20.

For lentiviral production, HEK293FT cells were co-transfected with pLKO constructs and packaging plasmids (VSVG, RSV-REV, and PMDLg/pPRE) using Lipofectamine 2000 (Invitrogen) as previously described 21. Stable clones were selected with puromycin. Overexpression was achieved using pcDNA-Flag, pcDNA-Flag-Gal-1, or pcDNA-Myc-SOX2 plasmids.

### Western blot analysis and immunoprecipitation

Cell lysates were prepared using RIPA buffer supplemented with protease inhibitors. Protein concentration was determined using the Bradford assay. Western blotting was performed using antibodies against galectin-1, SOX2, NANOG, β-actin (Santa Cruz Biotechnology, TX, USA), and Flag (Sigma-Aldrich). Signals were visualized using enhanced chemiluminescence. For immunoprecipitation, cell lysates were incubated with anti-galectin-1 or anti-SOX2 antibodies followed by protein A/G agarose beads (Santa Cruz Biotechnology). Normal mouse or rabbit IgG was used as a negative control. Immunocomplexes were analyzed by western blotting as described previously 22.

### RT-PCR

Total RNA was extracted using Isol-RNA Lysis Reagent (5prime, Gaithersburg, MD, USA). cDNA was synthesized from 1 μg total RNA using ReverTra Ace® qPCR RT Master Mix (TOYOBO, Tokyo, Japan). PCR amplification was performed using Ex Taq DNA Polymerase (TaKaRa) as described previously 23. Primer sequences are listed in Supplementary Table S3.

### Sphere forming culture

Cells were cultured in ultralow-attachment plates (Corning Costar, MA, USA) in Mammary Epithelial Basal Medium (Lonza, Basel, Switzerland) supplemented with B27 (Gibco), 20 ng/mL EGF, and 20 ng/mL FGF (PeproTech). Cells were seeded at 3,000 cells/mL. After 15 days, spheres larger than 50 μm in diameter were counted.

### Transwell migration and invasion assays

Migration and invasion assays were performed using 8.0 μm pore transwell chambers (Corning Costar). For migration assays, filters were coated with 0.5 mg/mL collagen type I. For invasion assays, filters were coated with Matrigel (1:15 dilution). Cells (1 × 10⁵) suspended in serum-free medium were seeded into the upper chamber. Medium containing 10% FBS was placed in the lower chamber. After 20 h incubation, migrated or invaded cells were fixed, stained with H&E, and counted in three random fields per membrane from three independent experiments.

### Side population analysis

Side population analysis was performed using the Hoechst 33342 dye exclusion method. MKN28 cells were transfected with scRNA or galectin-1 siRNA and harvested after 48 h. Cells (1 × 10⁶ per tube) were incubated in HBSS containing 2% FBS and 10 mM HEPES with Hoechst 33342 (5 μg/mL) in the presence or absence of reserpine (50 μM) at 37 °C for 60 min. Cells were washed with ice-cold buffer and stained with propidium iodide (2 μg/mL) before flow cytometric analysis.

### Luciferase reporter assay

AGS cells were co-transfected with the SOX2 reporter plasmid (pGL3-SRR2) and β-galactosidase plasmid for normalization. Forty-eight hours after transfection, luciferase activity was measured using a dual-luciferase reporter assay system as previously described 24.

### Xenograft experiments

Six-week-old female BALB/c nude mice (Orient Bio, Seoul, Korea) were subcutaneously injected with sphere-cultured AGS cells (1 × 10⁵) or YCC-2 cells (1 × 10³-1 × 10⁵). Mice were randomly assigned to groups, and tumor measurements were performed in a blinded manner. Tumor size was measured weekly and calculated as (a² × b × 0.5), where a represents the shorter diameter and b the longer diameter. Each experimental group consisted of five mice.

### Human gastric cancer tissues

Tissue microarrays were constructed using 2-mm cores from paraffin-embedded gastric carcinoma specimens (Superbiochips Laboratories, Seoul, Korea). Immunohistochemical analysis was performed as described previously 25.

### Immunohistochemistry

Paraffin-embedded gastric cancer tissues were deparaffinized and subjected to antigen retrieval in citrate buffer (Vector Laboratories, CA, USA) using microwave heating for 15 min. Endogenous peroxidase activity was blocked with 3% H₂O₂ for 15 min. Sections were incubated with 10% normal goat serum for 10 min and then with primary antibodies against galectin-1 (1:200) or SOX2 overnight at 4 °C. Detection was performed using the Vectastain® ABC kit (Vector Laboratories), as described previously 26, and visualized with diaminobenzidine (DAB). Slides were counterstained with hematoxylin.

### cDNA microarray

Microarray analysis was performed in AGS cells following galectin-1 knockdown as previously described 27. Differentially expressed genes were defined as those showing a linear fold change ≥ 2.0 (linear scale) with an adjusted p-value < 0.05.

### Statistical analysis

Data are expressed as mean ± SD or SEM as indicated. Comparisons between two groups were performed using Student's t-test. Multiple-group comparisons were performed using one-way ANOVA with appropriate post hoc tests. Survival analyses were conducted using the Kaplan-Meier method, and differences were assessed using the log-rank test. Associations between protein expression and clinicopathological variables were analyzed using the χ² test or Mann-Whitney U test as appropriate. Univariate and multivariate Cox proportional hazards models were used to calculate hazard ratios (HRs) and 95% confidence intervals (CIs). Statistical analyses were performed using SPSS version 29.0 (IBM, Chicago, IL, USA) and GraphPad Prism 8. A p-value < 0.05 was considered statistically significant.

## Results

### Inhibition of galectin-1 suppresses proliferation, motility, and stemness of gastric cancer cells

**Figure 1:**
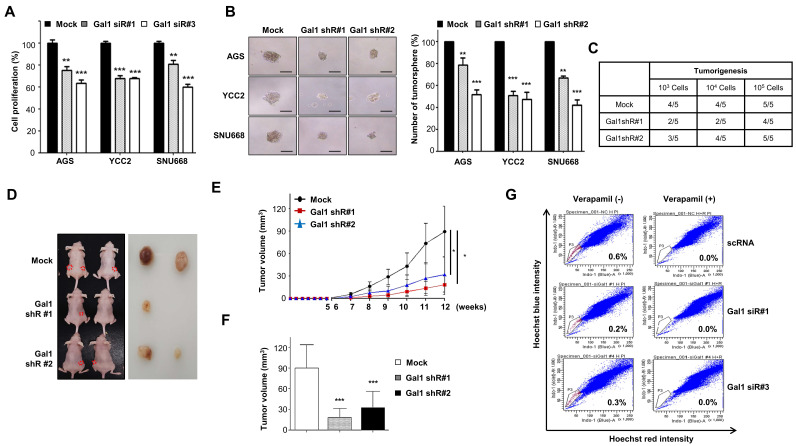
Inhibition of galectin-1 reduces tumorsphere formation, cell proliferation, and motility in gastric cancer cells. (A) Proliferation of AGS, YCC-2, and SNU-668 cells transfected with control siRNA (scRNA) or galectin-1 siRNAs (#1 and #3) was measured using a WST assay. (B) Tumorsphere formation assay following galectin-1 knockdown. Cells (5,000 cells/mL) were seeded in ultralow-attachment 24-well plates and cultured in sphere-forming medium for 2 weeks. Representative images (left) and quantification (right) are shown. Scale bar, 50 μm. (C-F) Tumorigenicity of galectin-1-depleted YCC-2 cells in nude mice. (C) Experimental design. (D) Representative tumor images. (E) Tumor growth curves over 12 weeks. (F) Final tumor weights at 12 weeks. (G) Side population analysis of MKN28 cells transfected with control or galectin-1 siRNA, performed as described in Materials and Methods. Data are presented as mean ± SD (n = 5 for *in vitro* assays; n = 5 mice per group for xenograft experiments). Statistical significance was determined using Student's t-test (*p < 0.05, **p < 0.01, ***p < 0.001).

We first examined galectin-1 expression across 11 gastric cancer cell lines by RT-PCR and western blotting (Suppl. Fig. S1). Among them, AGS, YCC-2, and SNU-668 cells exhibited relatively high levels of galectin-1 and were selected for further analysis. Transient knockdown of galectin-1 using siRNAs effectively reduced both mRNA and protein expression (Suppl. Fig. S2). Functional assays demonstrated that galectin-1 depletion significantly inhibited cell proliferation in AGS, YCC-2, and SNU-668 cells (Fig. 1A). In addition, galectin-1 knockdown markedly impaired cell motility, as evidenced by reduced migration and invasion in transwell assays (Suppl. Fig. S3).

We next assessed the effect of galectin-1 on stemness properties using a tumorsphere formation assay (Fig. 1B). The number and size of spheres significantly decreased in galectin-1-depleted cells compared with controls. To further validate the role of galectin-1 in cancer stemness both *in vitro* and *in vivo*, we established stable knockdown of galectin-1 in YCC-2 cells using shRNA (Suppl. Fig. S4A) and transient knockdown in MKN28 cells using siRNA (Suppl. Fig. S4B). Tumorsphere-derived YCC-2 cells were inoculated into nude mice to assess tumor-initiating capacity. While as few as 10,000 wild-type YCC-2 cells consistently generated tumors, galectin-1-depleted cells formed tumors in only 2-3 of 5 mice (Fig. 1C), and the resulting tumors displayed markedly slower growth and reduced volume compared with controls (Fig. 1D-F).

Finally, to determine whether galectin-1 affects the side population fraction, a surrogate marker of cancer stemness, we analyzed MKN28 cells following galectin-1 knockdown. The proportion of side population cells was significantly reduced in galectin-1-depleted cells (Fig. 1G). Together, these findings indicate that galectin-1 promotes proliferation, motility, and stem-like properties of gastric cancer cells in vitro and in vivo. MKN28 cells were employed specifically for side population (SP) analysis in both knockdown and overexpression settings because, under our Hoechst 33342 dye exclusion conditions, they display a well-resolved, reproducible SP fraction with substantially higher baseline SP percentages than other tested cell lines, enabling reliable and sensitive detection of galectin-1-dependent changes in the cancer stem-like population.

### Overexpression of galectin-1 enhances proliferation, motility, and stemness of gastric cancer cells

**Figure 2:**
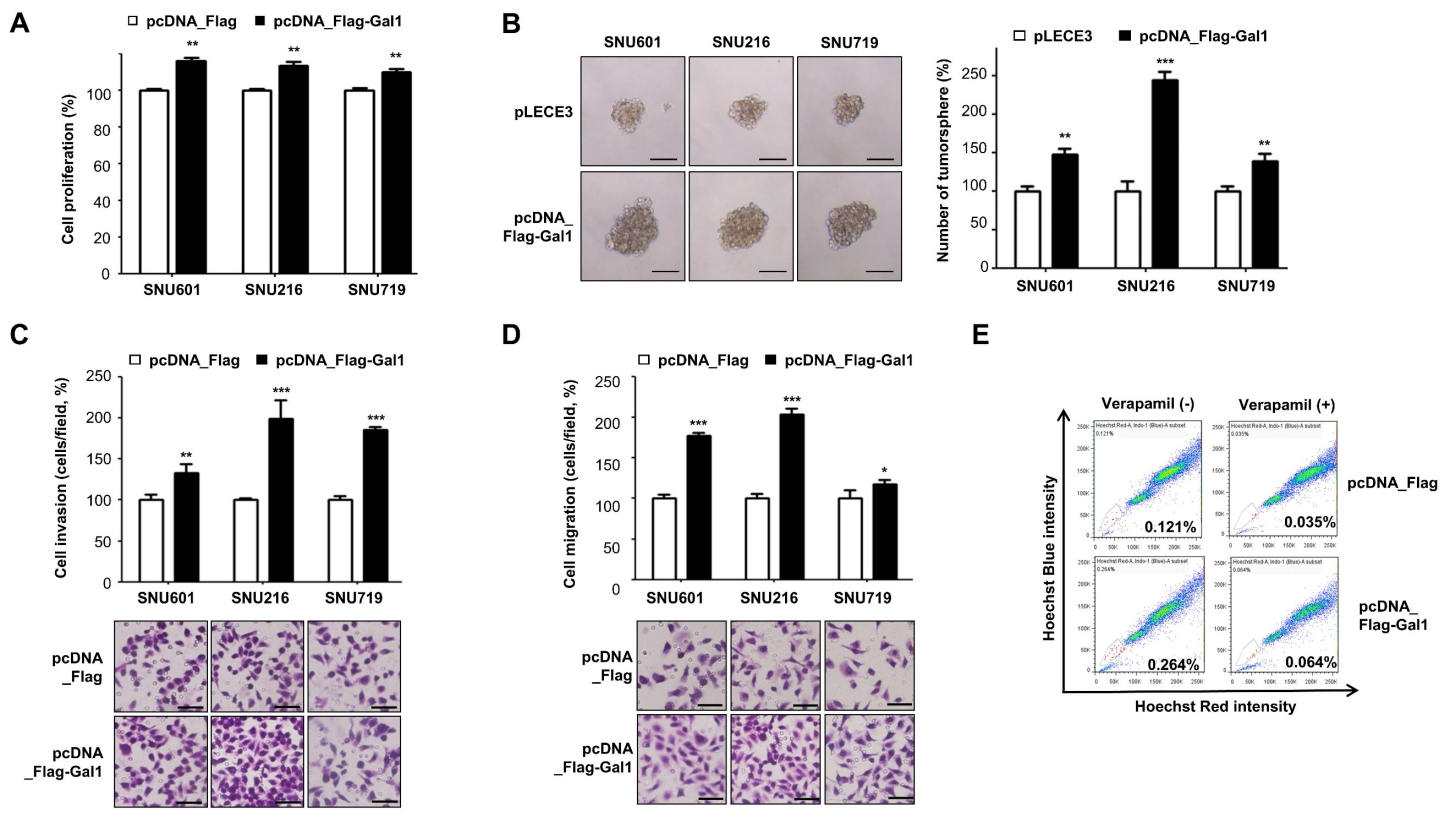
Overexpression of galectin-1 enhances tumorsphere formation, cell proliferation, and motility in gastric cancer cells. (A) Proliferation of SNU-601, SNU-216, and SNU-719 cells transfected with empty vector or galectin-1 expression vector was measured using a WST assay. (B) Tumorsphere formation assay after 2 weeks of culture. Cells were seeded at 5,000 cells/mL (500 μL per well) in ultralow-attachment 24-well plates. Representative images (left, 100×) and quantification (right) are shown. Scale bar, 50 μm. (C-D) Migration and invasion assays using transwell chambers. Representative images (bottom) and quantification (top) are shown. Five random fields per well were counted. Scale bar, 50 μm. (E) Side population analysis in MKN28 cells transfected with empty vector (pLECE3) or galectin-1 expression vector (pLECE3-Gal-1). Data are presented as mean ± SD (n = 5). Statistical significance was determined using Student's t-test (*p < 0.05, **p < 0.01, ***p < 0.001).

To further evaluate the role of galectin-1, we overexpressed galectin-1 in SNU-601, SNU-216, and SNU-719 gastric cancer cell lines, which lack detectable endogenous galectin-1 expression. Transfection with a galectin-1 expression vector resulted in robust overexpression, as confirmed by RT-PCR and western blotting (Suppl. Fig. S5A). Galectin-1 overexpression significantly promoted cell proliferation in all three cell lines compared with controls (Fig. 2A).

We next examined the impact of galectin-1 overexpression on cancer stemness. Tumorsphere formation assays demonstrated that enforced galectin-1 expression markedly increased both the number and size of tumorspheres (Fig. 2B). Consistent with these findings, galectin-1 overexpression enhanced cell migration and invasion as determined by transwell assays (Fig. 2C and D). Moreover, analysis of side population fractions revealed that galectin-1 overexpression increased the proportion of side population cells in MKN28 cells (Suppl. Fig. S5B and Fig. 2F). Collectively, these results indicate that galectin-1 overexpression reinforces cancer stem cell-like properties in gastric cancer cells, including enhanced proliferation, tumorsphere-forming ability, migration, and invasion.

### Galectin-1 regulates SOX2 expression and activity to promote stemness in gastric cancer cells

**Figure 3:**
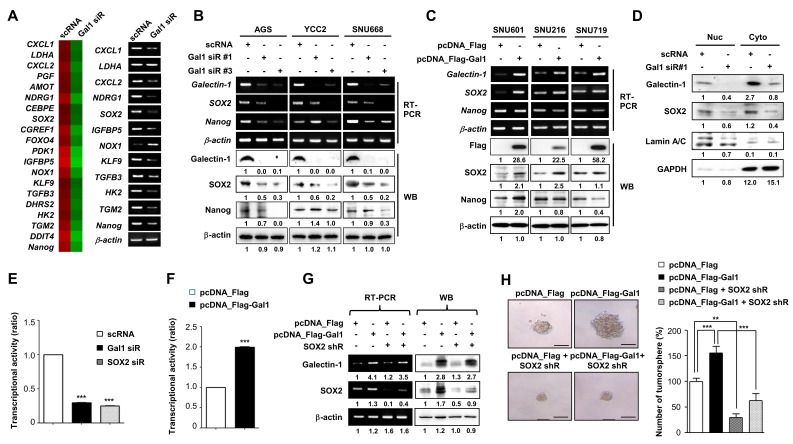
Galectin-1 regulates SOX2 expression and transcriptional activity to promote stemness in gastric cancer cells. (A) Microarray analysis of AGS cells transfected with galectin-1 siRNA for 48 h. Heatmap showing the 20 most significantly downregulated genes (left) and validation of 12 selected genes by RT-PCR (right). (B-C) mRNA and protein expression of galectin-1, SOX2, and NANOG following galectin-1 knockdown (siRNA) or overexpression (pcDNA-Flag-Gal-1) in six gastric cancer cell lines. (D) Nuclear-cytosolic fractionation analysis following galectin-1 knockdown. (E-F) SOX2 transcriptional activity measured using a luciferase reporter assay in AGS cells co-transfected with SOX2 reporter and galectin-1 shRNA or SOX2 shRNA (E), or galectin-1 expression vector (F). (G) Western blot analysis of galectin-1 and SOX2 in AGS cells transfected with galectin-1 expression vector alone or together with SOX2 shRNA. Densitometric quantification is shown below each blot. (H) Tumorsphere formation assay following galectin-1 overexpression with or without SOX2 knockdown. Representative images (left, 100×) and quantification (right) are shown. Scale bar, 50 μm. Data are presented as mean ± SD (n = 5). Statistical significance was determined using Student's t-test (**p < 0.01, ***p < 0.001).

To elucidate the mechanism by which galectin-1 regulates cancer stemness, proliferation, and motility, we performed microarray analysis following galectin-1 knockdown. Differential expression profiling revealed broad transcriptional changes across multiple pathways (Fig. 3A; Suppl. Table S1 and S2). Notably, the stemness-related transcription factors SOX2 and NANOG were among the most significantly downregulated genes. Consistently, galectin-1 knockdown reduced both mRNA and protein expression of SOX2 and NANOG, whereas galectin-1 overexpression increased their levels in gastric cancer cell lines (Fig. 3B and C). Interestingly, galectin-1 expression showed a strong correlation with SOX2, but not with NANOG, prompting us to focus further investigation on SOX2.

To define the relationship between galectin-1 and SOX2, we transfected AGS cells separately with galectin-1 siRNA or SOX2 siRNA. RT-PCR and western blotting confirmed that galectin-1 knockdown reduced SOX2 expression, whereas SOX2 knockdown did not alter galectin-1 levels (Suppl. Fig. S6A). Functional assays further demonstrated that knockdown of either galectin-1 or SOX2 individually decreased tumorsphere formation, migration, and invasion (Suppl. Fig. S6B-D). Conversely, galectin-1 overexpression increased SOX2 expression, whereas SOX2 overexpression did not affect galectin-1 levels (Suppl. Fig. S7A). Overexpression of either galectin-1 or SOX2 enhanced tumorsphere formation, cell motility, CD44 expression, and ALDH enzymatic activity, further supporting their roles in promoting stem-like malignant phenotypes (Suppl. Fig. S7B-D and S8). It should be noted that the increase in SOX2 protein expression upon galectin-1 overexpression (Fig. 7A, Suppl. Fig. S7A) reflects experimental overexpression conditions in cell line models, whereas Figure 8A presents a correlation analysis of endogenous mRNA expression levels across a large TCGA patient cohort. Given the transcriptional and post-translational complexity of SOX2 regulation in diverse tumor specimens, the correlation between galectin-1 and SOX2 mRNA in patient datasets may not directly parallel the acute protein-level changes observed under forced overexpression in a single cell line. Furthermore, galectin-1's primary effect on SOX2 appears to operate at the level of nuclear stability and transcriptional activity rather than exclusively at the level of total protein expression, which may further account for the differences observed between experimental overexpression and population-level transcriptomic correlations.

We next examined whether galectin-1 regulates SOX2 abundance and transcriptional activity. Subcellular fractionation showed that galectin-1 knockdown reduced the detectable levels of SOX2 in the nucleus (Fig. 3D). Furthermore, luciferase reporter assays using a SOX2-binding promoter sequence revealed that galectin-1 overexpression significantly increased SOX2 transcriptional activity, whereas knockdown of either galectin-1 or SOX2 suppressed it (Fig. 3E and F). These findings suggest that galectin-1 regulates the nuclear abundance and transcriptional function of SOX2.

We further investigated whether galectin-1 promotes stemness and motility through SOX2. We confirmed that overexpressed galectin-1 led to an increase in SOX2 expression, which was effectively reduced by subsequent SOX2 knockdown (Fig. 3G). Consequently, the galectin-1-induced enhancement of cell proliferation, tumorsphere formation, and motility was significantly suppressed by SOX2 depletion (Fig. 3H; Suppl. Fig. S9A-D). Taken together, these findings demonstrate that galectin-1 promotes the stemness, proliferation, and motility of gastric cancer cells by upregulating SOX2 expression and enhancing its nuclear abundance and transcriptional activity.

### *O*-GlcNAcylation-dependent interaction between galectin-1 and SOX2 regulates SOX2 activity

**Figure 4:**
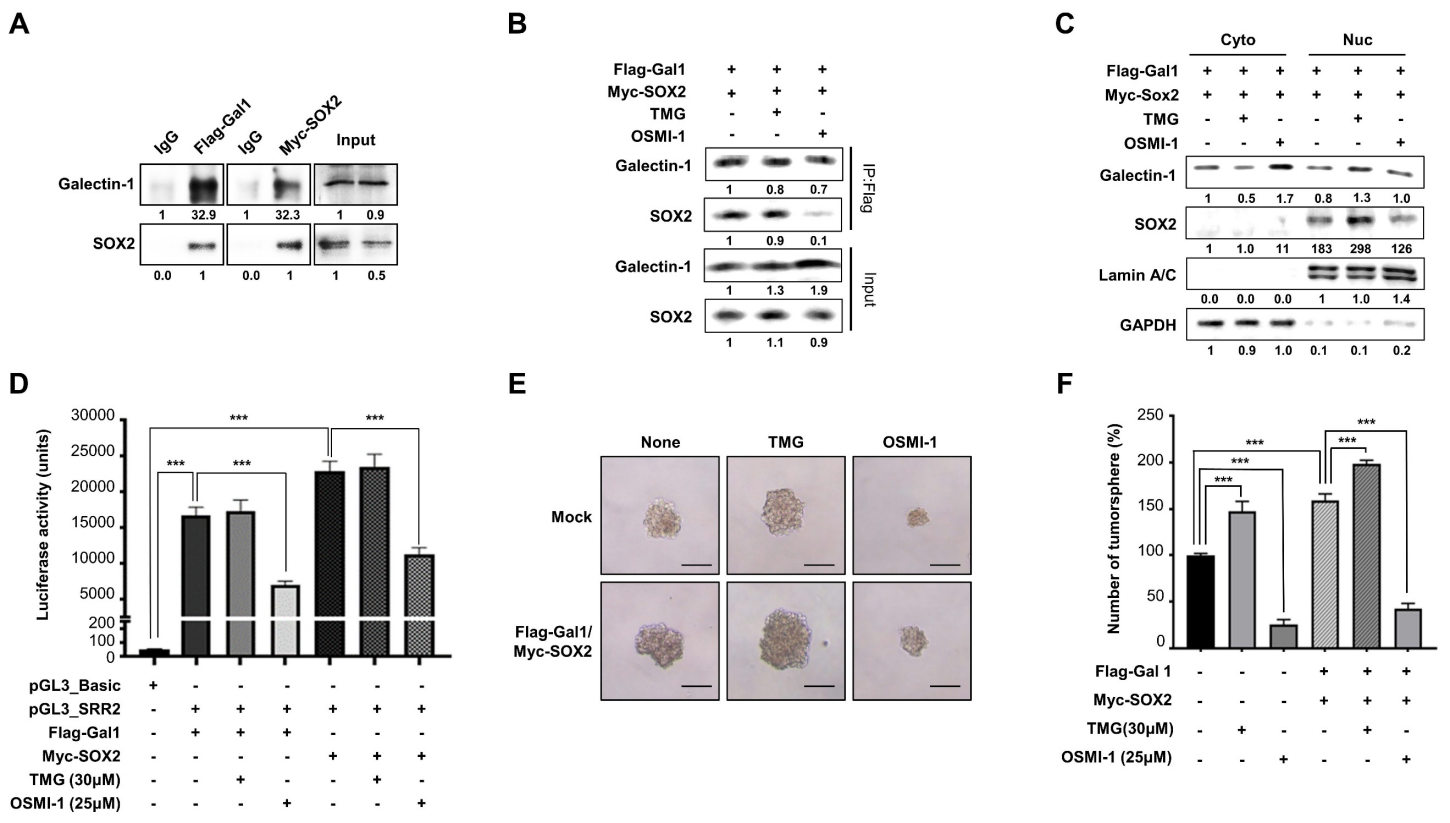
O-GlcNAcylation regulates the galectin-1-SOX2 interaction and contributes to stemness in gastric cancer cells. (A) Co-immunoprecipitation (IP) demonstrates interaction between Flag-Gal-1 and Myc-SOX2. (B) IP analysis showing that O-GlcNAc transferase inhibition (OSMI-1) reduces, whereas O-GlcNAcase inhibition (TMG) preserves, the galectin-1-SOX2 interaction. (C) Nuclear-cytosolic fractionation showing altered nuclear SOX2 detection following OSMI-1 or TMG treatment. Densitometric analysis is indicated below each blot. (D) Luciferase reporter assay demonstrating enhancement of SOX2 transcriptional activity by galectin-1 and its suppression by O-GlcNAcylation inhibition. (E-F) Tumorsphere formation assays showing attenuation of galectin-1-induced sphere formation upon O-GlcNAcylation inhibition. Representative images (E, 100×) and quantification (F) are shown. Scale bar, 50 μm. Data are presented as mean ± SD (n = 5). Statistical significance was determined using Student's t-test (***p < 0.001).

Previous studies have reported that galectin-1, despite lacking a canonical nuclear localization signal (NLS), can translocate into the nucleus through interactions with partner proteins 28. In contrast, SOX2 contains a defined NLS that mediates its nuclear import 29. Based on this, we examined whether galectin-1 physically interacts with SOX2. Co-immunoprecipitation (IP) assays confirmed an association between the two proteins (Fig. 4A). Confocal immunofluorescence analysis further demonstrated nuclear co-localization of galectin-1 and SOX2 (Suppl. Fig. S10), supporting their spatial association.

We next investigated whether this interaction functionally influences SOX2 nuclear abundance and transcriptional activity. To explore the basis of this association, we focused on the potential role of SOX2 *O*-GlcNAcylation, a modification previously reported to enhance SOX2 stability and transcriptional activity. 30. We therefore examined whether *O*-GlcNAcylation of SOX2 is critical for its interaction with galectin-1. We treated cells with OSMI-1, an *O*-GlcNAc transferase inhibitor, or TMG, an *O*-GlcNAcase inhibitor. IP analysis showed that the galectin-1-SOX2 interaction was significantly attenuated by OSMI-1 treatment, whereas it was preserved under TMG treatment (Fig. 4B). Consistent with this, nuclear fractionation demonstrated that nuclear SOX2 levels increased by TMG and decreased by OSMI-1 (Fig. 4C).

We next assessed whether galectin-1-mediated engagement of *O*-GlcNAcylated SOX2 affected its function. Luciferase reporter assays revealed that SOX2 transcriptional activity was enhanced in the presence of galectin-1, while inhibition of *O*-GlcNAcylation abrogated this effect (Fig. 4D). In addition, tumorsphere formation assay showed that galectin-1-induced sphere formation was diminished following *O*-GlcNAcylation inhibition (Fig. 4E and F). Taken together, these results indicate that galectin-1 interacts with SOX2 in an *O*-GlcNAcylation-dependent manner, thereby promoting its nuclear abundance and transcriptional activity. This mechanism contributes to the maintenance of stem-like properties in gastric cancer cells.

### *O*-GlcNAcylation-defective mutations in galectin-1 and SOX2 impair their interaction and reduce gastric cancer stemness

**Figure 5:**
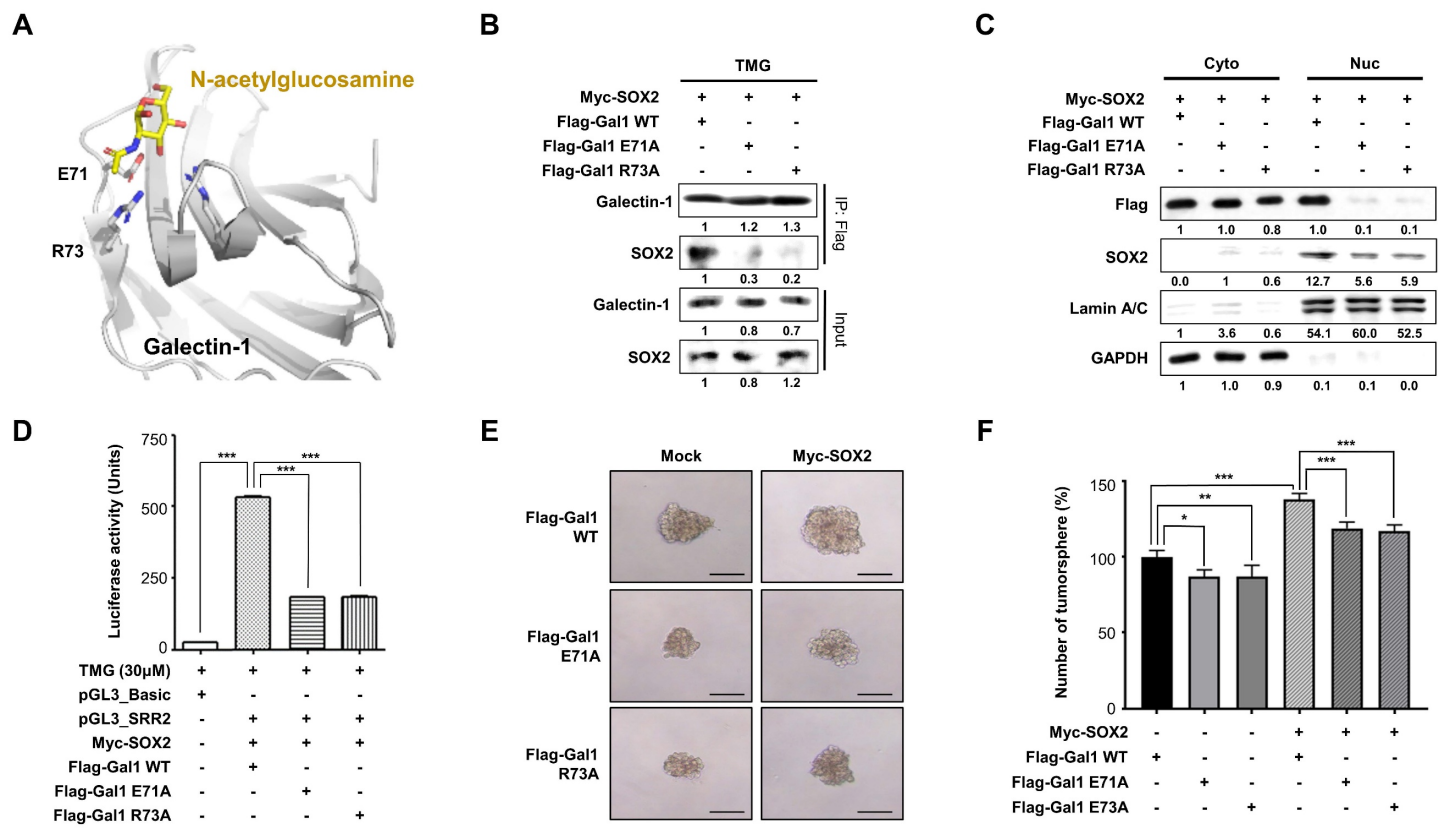
CRD mutations in galectin-1 impair SOX2 interaction and reduce gastric cancer stemness. (A) Structural modeling of human galectin-1 (PDB ID: 4Y1X) bound to N-acetylglucosamine identifying residues E71 and R73 as critical for carbohydrate-mediated recognition. (B) Co-immunoprecipitation showing reduced SOX2 binding to galectin-1 mutants (E71A and R73A) compared with wild type. (C) Nuclear-cytosolic fractionation demonstrating decreased nuclear SOX2 detection in cells expressing galectin-1 mutants. (D) Luciferase reporter assay assessing SOX2 transcriptional activity in the presence of wild-type or mutant galectin-1. (E-F) Tumorsphere formation assays in AGS cells expressing galectin-1 wild type or mutants. Representative images (E, 100×) and quantification (F) are shown. Scale bar, 50 μm. Data are presented as mean ± SD (n = 5). Statistical significance was determined using Student's t-test (*p < 0.05, **p < 0.01, ***p < 0.001).

Based on structural modeling of the galectin-1 sugar-binding site (PDB ID:4Y1X), we identified residues E71 and R73 as putative sites important for recognition of *O*-GlcNAcylated proteins (Fig. 5A). To validate their role, we generated galectin-1 mutants (E71A and R73A) and assessed their ability to interact with SOX2. Mutations at either residue markedly reduced galectin-1 -SOX2 interaction, as determined by IP (Fig. 5B), and concomitantly decreased the detectable levels of SOX2 in the nucleus (Fig. 5C). Functionally, these mutants exhibited reduced SOX2 transcriptional activity (Fig. 5D) and impaired tumorsphere formation (Fig. 5E and F). These results indicate that residues E71 and R73 in galectin-1 are individually critical for binding to *O*-GlcNAcylated SOX2 and for sustaining stemness properties, consistent with predictions from our structural modeling analysis. Furthermore, SOX2 immunoprecipitation followed by anti-*O*-GlcNAc immunoblotting revealed that expression of galectin-1 mutants (E71A or R73A) was associated with reduced co-detection of *O*-GlcNAc signal in SOX2 immunoprecipitants compared with wild-type galectin-1 (Suppl. Fig. S11), consistent with impaired carbohydrate-mediated engagement. These experiments were conducted in AGS cells co-transfected with Myc-SOX2 and either wild-type or mutant Flag-Gal-1. It should be noted that in this experimental design, SOX2 was immunoprecipitated using an anti-Myc antibody, and the galectin-1 mutants themselves were not captured in the pulldown; rather, their presence in the cellular milieu was associated with reduced O-GlcNAc co-immunoprecipitation with SOX2. This observation suggests that expression of CRD-deficient galectin-1 mutants may indirectly alter the O-GlcNAcylation status or accessibility of SOX2, possibly by competing with endogenous wild-type galectin-1 for SOX2 binding in an O-GlcNAc-dependent context.

**Figure 6:**
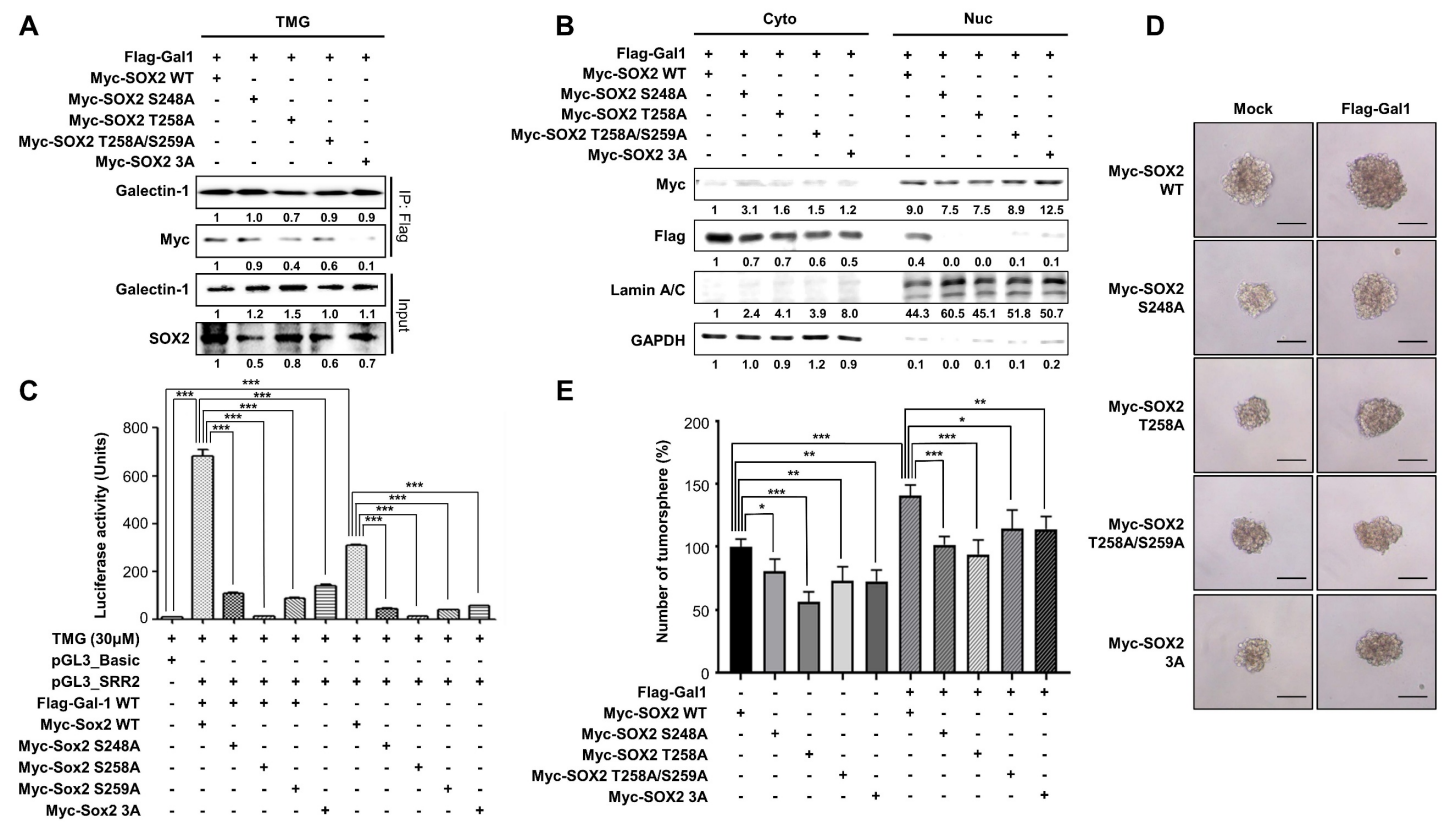
*O*-GlcNAcylation-deficient SOX2 mutants impair galectin-1 interaction and reduce gastric cancer stemness. (A) Co-immunoprecipitation showing reduced galectin-1 binding to SOX2 *O*-GlcNAc-deficient mutants (S248A, T258A, T258A/S259A, and 3A [S248A/T258A/S259A]) under TMG treatment. (B) Nuclear-cytosolic fractionation showing that SOX2 mutants remain detectable in the nuclear fraction, whereas galectin-1 is not detected in the nucleus when co-expressed with mutant SOX2. (C) Luciferase reporter assays demonstrating reduced transcriptional activity of SOX2 mutants compared with wild-type SOX2. (D-E) Tumorsphere formation assays showing decreased sphere-forming ability of SOX2 mutants. Representative images (D, 100×) and quantification (E) are shown. Scale bar, 50 μm. Data are presented as mean ± SD (n = 5). Statistical significance was determined using Student's t-test (*p < 0.05, **p < 0.01, ***p < 0.001).

We next investigated whether *O*-GlcNAcylation of SOX2 is required for its interaction with galectin-1 and the maintenance of stemness. Previous studies identified *O*-GlcNAcylation sites within the amino acid region 248-264 of SOX2 31. Accordingly, we employed SOX2 *O*-GlcNAcylation-deficient mutants (S248A, T258A, T258/S259A, and the triple mutant S248A/T258A/S259A [3A]). Co-immunoprecipitation analysis revealed that each single mutation was sufficient to weaken the interaction between SOX2 and galectin-1, and the triple mutant completely abolished this binding (Fig. 6A). Subcellular fractionation further demonstrated that, even when SOX2 mutants were overexpressed, galectin-1 was not detected in the nuclear fraction, whereas SOX2 itself remained detectable in the nucleus, suggesting SOX2 *O*-GlcNAcylation affects its ability to bind galectin-1 but does not alter the cellular localization of SOX2 (Fig. 6B). Consistent with the reduced interaction, all SOX2 mutants exhibited attenuated transcriptional activity compared with wild-type SOX2 (Fig. 6C). Functionally, tumorsphere assays showed that each mutant markedly decreased sphere-forming capacity, with the triple mutant producing the most pronounced reduction (Fig. 6D and E). Taken together, these results demonstrate that *O*-GlcNAcylation at residues S248, T258, and S259 is critical for SOX2 binding to galectin-1 and for sustaining its transcriptional activity and stem-like properties but does not govern its nuclear import.

### *In vivo* validation of the galectin-1/SOX2 axis and clinical relevance of galectin-1 in gastric cancer

**Figure 7:**
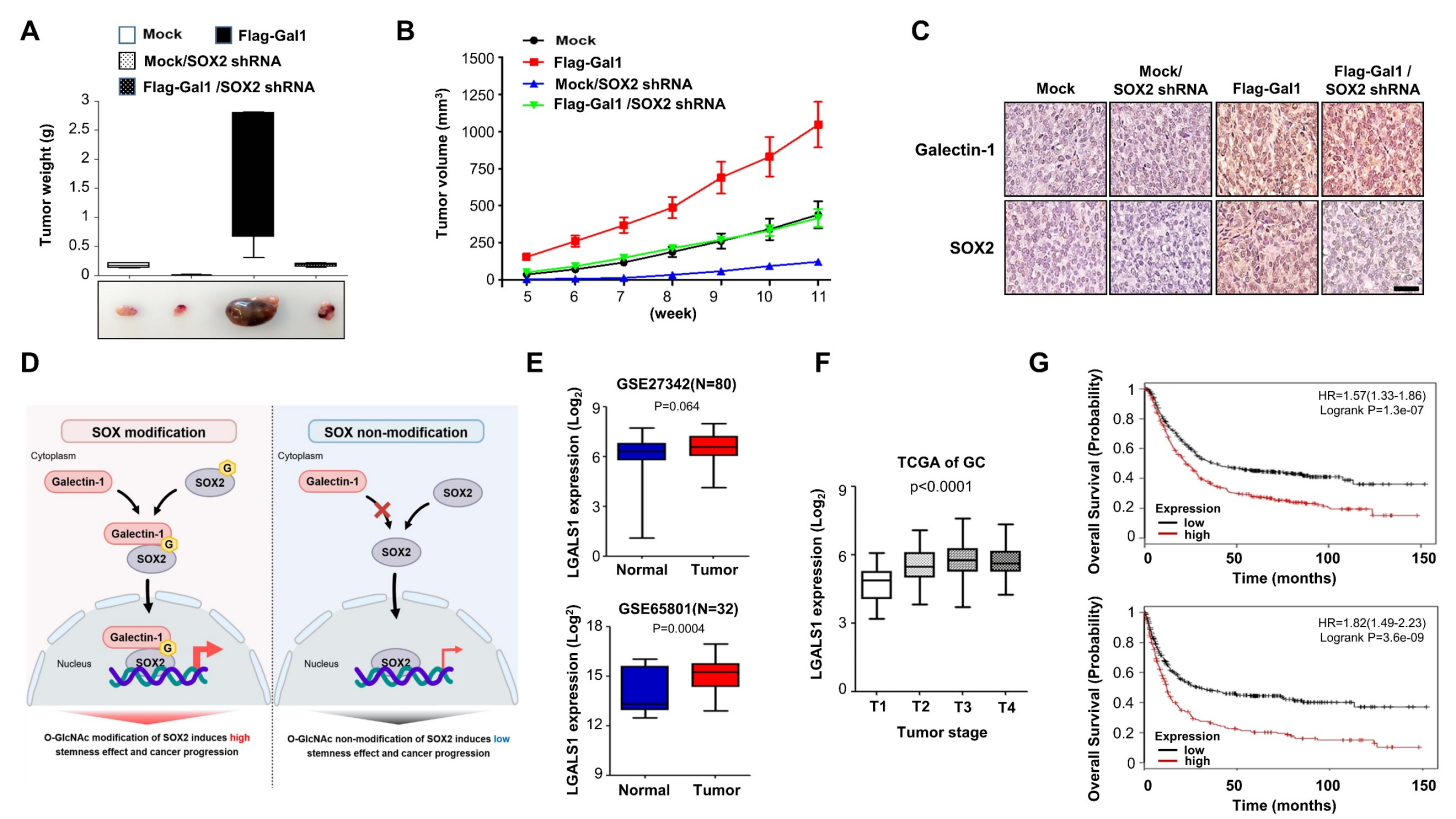
*In vivo* validation of the galectin-1/SOX2 axis and clinical relevance of galectin-1 in gastric cancer. (A-C) Xenograft assays using AGS cells stably overexpressing galectin-1 with or without SOX2 knockdown. (A) Representative tumor photographs (bottom) and tumor weights at 12 weeks (top). (B) Tumor growth curves measured over 12 weeks. (C) Immunohistochemical staining of xenografted tumors for galectin-1 and SOX2. Scale bars, 400 μm. (D) Schematic model illustrating *O*-GlcNAcylation-dependent galectin-1/SOX2 interaction promoting stemness-driven tumor progression. (E) Galectin-1 expression in gastric tumor tissues compared with normal tissues in GEO datasets (GSE27342, GSE65801). (F) Association between galectin-1 expression and TNM stage in TCGA gastric cancer cohort (n = 265). (G) Kaplan-Meier survival analysis showing that high galectin-1 expression correlates with shorter overall survival (OS) and earlier first progression (FP).

To validate our in vitro findings in vivo, we established a xenograft model using AGS gastric cancer cells stably overexpressing galectin-1 with or without SOX2 knockdown (Suppl. Fig. S8A). Galectin-1 overexpression markedly promoted tumor formation, resulting in increased tumor size and accelerated tumor growth compared with control cells. This was confirmed by tumor weight measurements and representative tumor photographs (Fig. 7A), as well as by tumor growth curves (Fig. 7B). Importantly, concomitant depletion of SOX2 significantly attenuated tumor size and growth rate, even in the presence of galectin-1 overexpression. Immunohistochemical staining of xenografted tumors further demonstrated concordant expression patterns of galectin-1 and SOX2 (Fig. 7C). Together, these findings support that galectin-1 enhances gastric tumorigenesis *in vivo* in a SOX2-dependent manner. A schematic model summarizing the proposed mechanism is presented in Figure 7D.

We next examined the independent clinical relevance of galectin-1 in gastric cancer. Analysis of two independent GEO patient cohorts (GSE27342, n = 80, p = 0.002; GSE65801, n = 32, p = 0.0004) revealed significantly higher galectin-1 expression in tumor tissues compared with adjacent normal tissues (Fig. 7E). Consistent with this, TCGA gastric cancer samples (n = 265) showed that galectin-1 expression positively correlated with tumor TNM stage (Fig. 7F). Kaplan-Meier plotter analysis further showed that high galectin-1 expression was strongly associated with poor patient outcomes, including shorter overall survival (n = 876, p = 1.3 X 10^-7^) and earlier first progression (n = 641, p = 306 X 10^-9^) (Fig. 7G).

### Correlation of galectin-1 and SOX2 expression with overall survival and clinicopathological characteristics in gastric cancer patients

**Figure 8:**
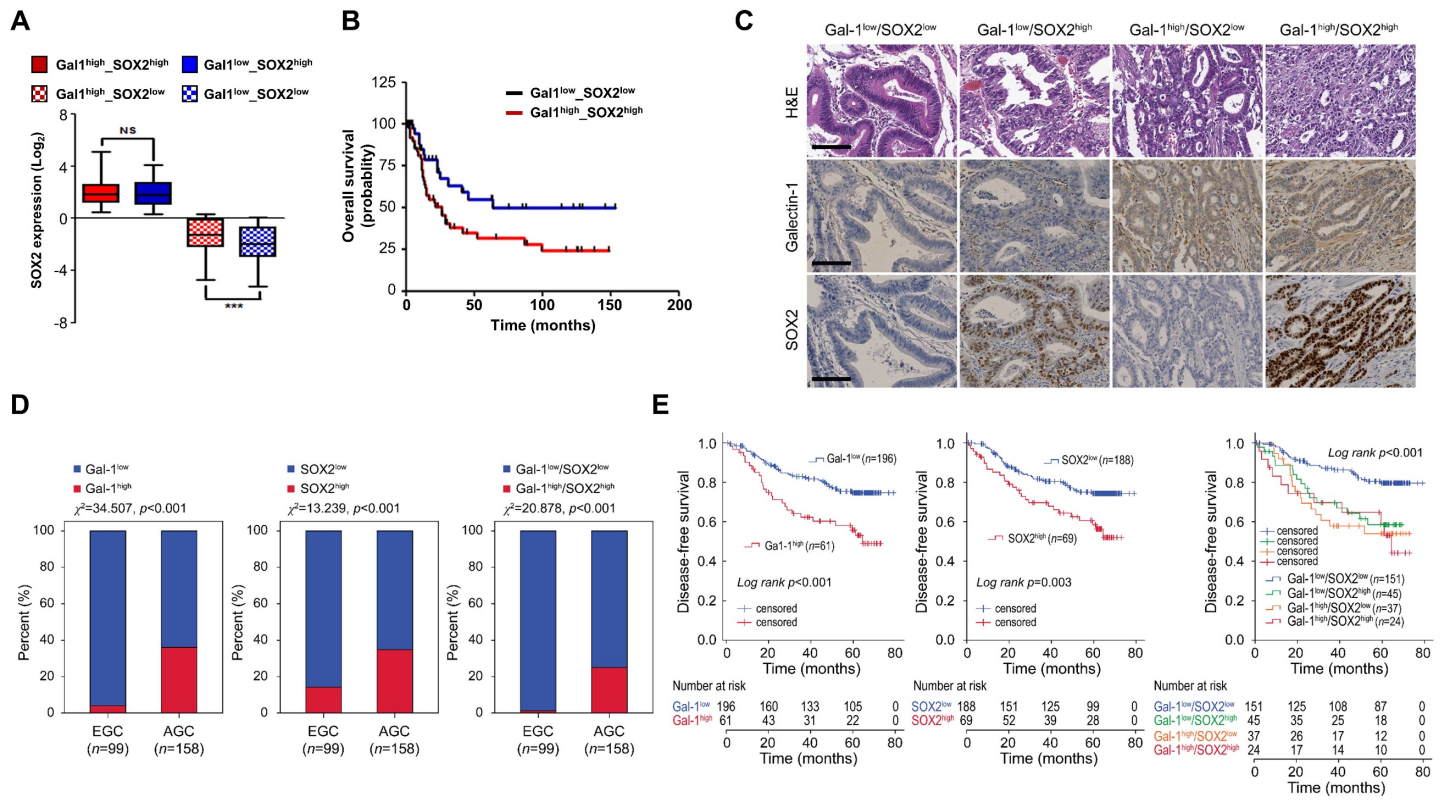
Correlation of galectin-1 and SOX2 expression with survival and clinicopathological characteristics in gastric cancer patients. (A) Correlation between galectin-1 and SOX2 expression in TCGA gastric cancer dataset. (B) Kaplan-Meier survival analysis of GSE15459 (n = 94) showing significantly reduced overall survival in the Gal-1^high^/SOX2^high^ group (χ² = 4.770, p = 0.0290). (C) Representative H&E, galectin-1, and SOX2 staining in human gastric cancer tissues. (D) Distribution of galectin-1 and SOX2 expression in early gastric cancer (EGC) and advanced gastric cancer (AGC). χ² test; p < 0.001. (E) Kaplan-Meier analysis of disease-free survival stratified by galectin-1 (p < 0.001), SOX2 (p = 0.003), or combined Gal-1/SOX2 expression (p < 0.001).

To determine whether the galectin-1/SOX2 axis has clinical significance, we analyzed co-expression patterns in patient cohorts. We first analyzed gene expression signatures in TCGA gastric cancer datasets and found a significant correlation between galectin-1 and SOX2 expressions (Fig. 8A). Kaplan-Meier survival analysis of a subset of 94 gastric cancer patients (GSE15459) further revealed that patients with high co-expression of galectin-1 and SOX2 had significantly reduced overall survival compared with other groups (p = 0.0290) (Fig. 8B). These data suggest a close association between the galectin-1/SOX2 axis and poor clinical outcomes in gastric cancer.

To corroborate these findings, we assessed galectin-1 and SOX2 protein expression by immunohistochemistry in a cohort of 257 gastric cancer specimens (Fig. 8C; Suppl. Fig. S12). High galectin-1 (Gal-1^high^) and SOX2 (SOX2^high^) expression were detected in 23.7% (61/257) and 26.8% (69/257), respectively. Gal-1^high^ tumors exhibited a strong association with adverse pathological features, including higher pT classification, lymph node metastasis, lymphovascular invasion, and advanced disease stage (Suppl. Table S4). Similarly, SOX2^high^ expression correlated with these same aggressive characteristics (Suppl. Table S4).

High expression of galectin-1, SOX2, or their combined elevation was significantly enriched in advanced gastric cancer compared with early-stage tumors (all χ^2^ tests, *p* < 0.001; Fig. 8D). Analysis of the prognostic impact showed that patients with Gal-1^high^ tumors had significantly poorer DFS than those with Gal-1^low^ tumors (Fig. 8E). SOX2^high^ expression was likewise associated with reduced DFS. Notably, concurrent Gal-1^high^/SOX2^high^ expression identified a subgroup with the poorest outcomes (DFS rate 54.2%) compared with patients exhibiting low expression of both markers (DFS rate 82.1%) (Fig. 8E). Univariate Cox regression analysis indicated that advanced pT stage, lymph node metastasis, Gal-1^high^, SOX2^high^, and the combined Gal-1^high^/SOX2^high^ profile were significantly associated with shortened DFS. In multivariate models, advanced pT stage, lymph node metastasis, Gal-1^high^, and combined Gal-1^high^/SOX2^high^ expression remained independent predictors of poor DFS (Suppl. Table S5). Together, these data demonstrate that elevated galectin-1 and SOX2 expression levels are strongly associated with aggressive clinicopathological features and adverse prognosis.

## Discussion

Here, we identify galectin-1 as an intracellular effector that selectively recognizes *O*-GlcNAcylated SOX2 and enhances its nuclear abundance and transcriptional competence, thereby sustaining cancer stemness. Intracellular galectin-1 associates with the stemness factor SOX2, highlighting a novel nuclear role beyond its classical extracellular functions.

Galectin-1 has been classically defined as a β-galactoside-binding lectin that binds glycans on cell-surface receptors and extracellular matrix components to regulate cell adhesion, migration, angiogenesis, and immune evasion 32. More recently, multiple galectin family members, including galectin-1, have been detected in the cytosol and nucleus, suggesting distinct intracellular roles 1, 33. Nevertheless, these intracellular functions have generally been considered carbohydrate-independent, as the carbohydrate recognition domain (CRD) was thought to primarily engage extracellular glycans 3, 33.

Our data extend this paradigm by demonstrating that galectin-1 recognizes an intracellular post-translational modification. Structural modeling and mutational analyses identified E71 and R73 within the CRD as essential residues for binding to *O*-GlcNAcylated SOX2. In support of this interpretation, previous studies have shown that mutation of the corresponding residue in galectin-3 (R74S) disrupts glycoprotein binding, underscoring the conserved functional importance of this arginine residue within the CRD 34. Disruption of these CRD residues or mutation of *O*-GlcNAc sites on SOX2 impaired their interaction and attenuated stemness phenotypes, supporting a reader-like role for galectin-1 in *O*-GlcNAc-dependent transcriptional regulation.

*O*-GlcNAcylation is a dynamic and reversible modification enriched in nuclear and cytoplasmic proteins and frequently elevated in cancer, linking metabolic reprogramming to malignant progression 35, 36. SOX2 is among the transcription factors known to undergo *O*-GlcNAcylation 37. This modification has been mapped to its C-terminal transactivation domain rather than its NLS or HMG DNA-binding domain 38 and *O*-GlcNAc-deficient SOX2 mutants exhibit reduced transcriptional activity and impaired stemness programs 30, 39. Within this framework, our findings demonstrate that galectin-1 selectively recognizes *O*-GlcNAcylated SOX2 and enhances its nuclear abundance and transcriptional competence without affecting intrinsic nuclear import. These results position galectin-1 as an intracellular effector linking *O*-GlcNAc-dependent signaling to SOX2-mediated stemness programs, thereby connecting metabolic regulation with transcriptional control in cancer. It is worth noting that galectin-1 is typically expressed at substantially higher molar concentrations than SOX2 within cells. SOX2 is a relatively low-abundance transcription factor, and it is therefore plausible that only a small fraction of the total galectin-1 pool engages with O-GlcNAc-modified SOX2 in the nucleus, while the bulk of galectin-1 may participate in other intracellular or extracellular functions. This stoichiometric consideration does not diminish the biological significance of the interaction, as transcription factor complexes are often functionally operative at sub-stoichiometric levels relative to their binding partners. Nevertheless, future quantitative proteomics or proximity ligation approaches may help define the fraction of nuclear galectin-1 that is engaged with SOX2 under physiological conditions.

The identification of galectin-1 as an intracellular effector recognizing *O*-GlcNAcylated SOX2 has important biological implications. By coupling a nutrient-sensitive post-translational modification to a key transcription factor, the galectin-1/SOX2 axis provides a mechanistic connection between metabolic signaling and transcriptional regulation in cancer. These findings expand the functional scope of galectin-1 beyond its classical extracellular roles and highlight this interaction as a potential therapeutic target.

The biological relevance of this axis is further supported by clinical observations. Galectin-1 expression was significantly elevated in gastric tumors, correlated with advanced stage, and predicted poor overall survival. Moreover, high co-expression of galectin-1 and SOX2 identified a subgroup of patients with particularly adverse outcomes. Although these clinical analyses are correlative, they are consistent with our mechanistic findings linking coordinated galectin-1/SOX2 expression to enhanced tumorigenic potential.

Several limitations should be acknowledged. Most mechanistic experiments were conducted in gastric cancer models, and whether this regulatory mechanism extends to other tumor types remains to be determined. In addition, although our data indicates that galectin-1 enhances nuclear abundance and activity of SOX2, the relative contributions of protein stability versus nuclear retention require further investigation. Future studies should also explore whether galectin-1 interacts with other *O*-GlcNAc-modified transcriptional regulators. Furthermore, the co-immunoprecipitation experiments in this study were performed without the use of cell-permeable chemical cross-linkers prior to cell lysis. Although the galectin-1-SOX2 interaction was consistently supported by multiple independent approaches including pharmacological perturbation and mutagenesis, the use of in-cell cross-linking strategies in future studies may further strengthen evidence for pre-existing protein complexes formed within intact cells. Additionally, residues E71 and R73, identified as critical for the galectin-1-SOX2 interaction, overlap with the LacNAc-binding site within the carbohydrate recognition domain. Consequently, mutation of these residues may affect multiple binding functions of the CRD, and the observed loss of SOX2 interaction cannot be attributed solely to disruption of O-GlcNAc recognition. Future studies incorporating competitive sugar inhibition assays or structural analyses of the galectin-1-SOX2 complex will be necessary to more precisely define the molecular basis of this interaction.

In conclusion, we identify galectin-1 as an intracellular effector that recognizes *O*-GlcNAcylated SOX2 and sustains stemness-associated transcriptional programs. The galectin-1/SOX2 axis provides a mechanistic link between *O*-GlcNAc signaling and cancer stemness and represents a potential therapeutic target in gastric cancer.

## Supplementary Material

Supplementary figures and tables.

## Data Availability

All data can be obtained by contacting the corresponding author.
