# Supplementary material for: Nuclear Galectin-1 Drives Cancer Progression through O-GlcNAcylation-Dependent Regulation of SOX2
Source: Int J Biol Sci. 2026 Apr 16;22(9):4584–97. doi: 10.7150/ijbs.124928 (PMC13182240; doi:10.7150/ijbs.124928)
Supplement: Supplementary file 1 — Supplementary figures and tables. [file ijbsv22p4584s1.pdf]

# Supplementary information

## Supplementary Tables

**Supplementary Table S1. Upregulated genes in AGS cells following galectin-1 silencing** Transcriptomic profiling was performed using microarray analysis in AGS cells transfected with galectin-1 siRNA. Genes significantly upregulated compared with control are listed. Differentially expressed genes were defined using a cut-off of $|\text{fold change}| \geq 2.0$ (linear scale) and adjusted $p < 0.05$.

| Gene Symbol | Gene Name | Fold Change (linear) |
|---|---|---|
| **Response to wounding** | | |
| CXCL11 | chemokine (C-X-C motif) ligand 11 | 2 |
| CXCL9 | chemokine (C-X-C motif) ligand 9 | 2 |
| CTGF | connective tissue growth factor | 2 |
| ENG | endoglin | 2 |
| **Biological adhesion** | | |
| CDH15 | cadherin 15, type 1, M-cadherin | 2 |
| TGFBI | transforming growth factor, beta-induced | 3.3 |
| BMP1 | bone morphogenetic protein 1 | 2 |
| **Cell motility** | | |
| CXCL12 | chemokine (C-X-C motif) ligand 12 | 2.5 |
| NRP2 | neuropilin 2 | 2.5 |
| **Cell migration** | | |
| CDH2 | cadherin 2, type 1, N-cadherin | 2 |
| DRD2 | dopamine receptor D2 | 2 |
| **Immune response** | | |
| IL1F10 | interleukin 1 family, member 10 | 2 |
| IL1RN | interleukin 1 receptor antagonist | 2 |
| **Positive regulation of signal transduction** | | |
| LGALS9 | lectin, galactoside-binding, soluble, 9 | 2 |

**Supplementary Table S2. Downregulated genes in AGS cells following galectin-1 silencing** Transcriptomic profiling was performed using microarray analysis in AGS cells transfected with galectin-1 siRNA. Genes significantly downregulated compared with control are listed. Differentially expressed genes were defined using a cut-off of |fold change| ≥ 2.0 and adjusted $p < 0.05$.

| Gene Symbol | Gene Name | Fold Change (linear) |
|---|---|---|
| **Response to wounding** | | |
| NOX1 | NADPH oxidase 1 | -2 |
| CXCL1 | chemokine (C-X-C motif) ligand 1 | -2 |
| CXCL2 | chemokine (C-X-C motif) ligand 2 | -2 |
| TGFB3 | transforming growth factor, beta 3 | -2 |
| **Blood vessel morphogenesis** | | |
| AMOT | angiomotin | -2 |
| PGF | placental growth factor | -2 |
| **Glucose metabolic process** | | |
| HK2 | hexokinase 2 pseudogene; hexokinase 2 | -2 |
| LDHA | lactate dehydrogenase A | -2 |
| PDK1 | pyruvate dehydrogenase kinase, isozyme 1 | -2.5 |
| **Cell activation** | | |
| NDRG1 | N-myc downstream regulated 1 | -2.5 |
| **Defense response** | | |
| CEBPE | CCAAT/enhancer binding protein (C/EBP), epsilon | -2 |
| **Regulation of cell proliferation** | | |
| SOX2 | SRY (sex determining region Y)-box 2 | -2 |
| CGREF1 | cell growth regulator with EF-hand domain 1 | -2.5 |
| DHRS2 | dehydrogenase/reductase (SDR family) member 2 | -2 |
| FOXO4 | forkhead box O4 | -2 |
| TGM2 | transglutaminase 2 | -2 |
| NANOG | Nanog homeobox pseudogene 8; Nanog homeobox | -2.5 |
| **Negative regulation of signal transduction** | | |
| DDIT4 | DNA-damage-inducible transcript 4 | -2.5 |
| IGFBP5 | insulin-like growth factor binding protein 5 | -2.5 |
| KLF9 | Kruppel-like factor 9 | -2 |

**Supplementary Table S3. Primer sequences used in this study**

| Gene name | Forward primer (5′→3′) | Reverse primer (5′→3′) |
|---|---|---|
| ***LGALS1*** | CTCTCGGGTGGAGTCTTCTG | ACGAAGCTCTTAGCGTCAGG |
| ***SOX2*** | AAAACAGCCCGGACCGCGTC | CTCGTCGATGAACGGCCGCT |
| ***NANOG*** | GGCGTCGGGATCGGATAAATA | GAAGAGGAGAGACAGTCTCCGT |
| ***CXCL9*** | GCAAGGAACCCCAGTAGTGA | TTTGGCTGACCTGTTTCTCC |
| ***CXCL12*** | TCAGCCTGAGCTACAGATGC | CTTTAGCTTCGGGTCAATGC |
| ***BMP1*** | GGAGAGACCCTGCAAGACAG | CGATAGGCTCAGGGAGTTTG |
| ***CDH2*** | GACAATGCCCCTCAAGTGTT | CCATTAAGCCGAGTGATGGT |
| ***LGALS9*** | CCTTTGACCTCTGCTTCCTG | AAACAGACAGGCTGGGAGAA |
| ***TGFB1*** | AACCCACAACGAAATCTATG | GTGCTGCTCCACTTTTAACT |
| ***NRP2*** | TCCACTGCTGACAAGGTTTG | ACTGGGGCTCCAGAGGTATT |
| ***CXCL1*** | AGGGAATTCACCCCAAGAAC | CACCAGTGAGCTTCCTCCTC |
| ***LDHA*** | TGGGAGTTCACCCATTAAGC | AGCACTCTCAACCACCTGCT |
| ***NDRG1*** | CGAGAGCTACATGACGTGGA | AAGAGGGGGTTGTAGCAGGT |
| ***IGFBP5*** | AAGAAGCTGACCCAGTCCAA | GAATCCTTTGCGGTCACAAT |
| ***NOX1*** | GGATGATCGTGACTCCCACT | TTTGGATGGGTGCATAACAA |
| ***KLF9*** | TCGCGTTTACAAGTGTCGAG | CTTCAGCTTCGGTTTTCCAG |
| ***TGFB3*** | CTATATCGGTGGCAAGAATC | ATCACCTCGTGAATGTTTTC |
| ***HK2*** | CCACCTTTGTGAGGTCCACT | GTCCTCAGGGATGGCATAGA |
| ***TGM2*** | GGGGTGAGAGAGGAAAGACC | TGCAGTCTAGGGAGCTGGAT |

**Supplementary Table S4.** Association of Galectin-1 and SOX2 expression with clinicopathologic characteristics in gastric cancer

| Category | Galectin-1, No. (%) | | | SOX2, No. (%) | | |
|---|---|---|---|---|---|---|
| | Low (*n*=196) | High (*n*=61) | *p* value | Low (*n*=188) | High (*n*=69) | *p* value |
| Age | 55.8 ± 12.3 | 59.7 ± 10.9 | 0.029* | 56.6 ± 12.4 | 57.3 ± 11.4 | 0.688 |
| Gender | | | 0.269 | | | 0.365 |
| Female | 65 (33.2) | 15 (24.6) | | 62 (33.0) | 18 (26.1) | |
| Male | 131 (66.8) | 46 (75.4) | | 126 (67.0) | 51 (73.9) | |
| Differentiation | | | <0.001* | | | 0.304 |
| Well | 27 (13.8) | 1 (1.6) | | 22 (11.7) | 6 (8.7) | |
| Moderate | 51 (26.0) | 28 (45.9) | | 61 (32.5) | 18 (26.1) | |
| Poor | 78 (39.8) | 30 (49.2) | | 73 (38.8) | 35 (50.7) | |
| Signet-ring cell | 35 (17.9) | 1 (1.6) | | 28 (14.9) | 8 (11.6) | |
| Mucinous | 5 (2.5) | 0 | | 4 (2.1) | 1 (1.4) | |
| Others | 0 | 1 (1.6) | | 0 | 1 (1.4) | |
| pT stage | | | <0.001* | | | 0.001* |
| pT1 | 95 (48.5) | 4 (6.6) | | 85 (45.2) | 14 (20.3) | |
| pT2 | 19 (9.7) | 19 (31.1) | | 28 (14.9) | 10 (14.5) | |
| pT3 | 47 (24.0) | 26 (42.6) | | 47 (25.0) | 26 (37.7) | |
| pT4 | 35 (17.8) | 12 (19.7) | | 28 (14.9) | 19 (27.5) | |
| LN metastasis | | | 0.012* | | | 0.015* |
| absent | 109 (55.6) | 22 (36.1) | | 105 (55.9) | 26 (37.7) | |
| present | 87 (44.4) | 39 (63.9) | | 83 (44.1) | 43 (62.3) | |
| LVI | | | 0.016* | | | 0.001* |
| absent | 132 (67.3) | 30 (49.2) | | 130 (69.1) | 32 (46.4) | |
| present | 64 (32.7) | 31 (50.8) | | 58 (30.9) | 37 (53.6) | |
| Depth of invasion | | | <0.001* | | | <0.001* |
| EGC | 95 (48.5) | 4 (6.6) | | 85 (45.2) | 14 (20.3) | |
| AGC | 101 (51.5) | 57 (93.4) | | 103 (54.8) | 55 (79.7) | |

LN, lymph node; LVI, lymphovascular invasion; EGC, early gastric cancer; AGC, advanced gastric cancer

*Statistically significant ($p < 0.05$)

**Supplementary Table S5.** Cox proportional univariate and multivariate analyses of disease-free survival in patients with gastric cancer

| Variables | Univariate analysis | | Multivariate analysis | | Multivariate analysis (including dual Gal-1/SOX2) | |
|---|---|---|---|---|---|---|
| | HR [95% CI] | *p* value | HR [95% CI] | *p* value | HR [95% CI] | *p* value |
| Gender (Male) | 1.115 [0.665-1.870] | 0.681 | | | | |
| Differentiation | 1.259 [0.994-1.595] | 0.563 | | | | |
| pT | 2.910 [2.219-3.817] | <0.001* | 2.524 [1.836-3.470] | <0.001* | 2.516 [1.833-3.454] | <0.001* |
| pN | 7.858 [4.023-15.348] | <0.001* | 3.078 [1.536-6.170] | 0.002* | 3.082 [1.537-6.179] | 0.002* |
| Gal-1$^{high}$ | 2.282 [1.412-3.687] | 0.005* | 2.086 [1.242-3.502] | 0.005* | | |
| SOX2$^{high}$ | 1.994 [1.238-3.210] | 0.001* | 1.356 [0.840-2.190] | 0.212 | | |
| Gal-1$^{high}$/SOX2$^{high}$ | 1.499 [1.230-1.827] | <0.001* | | | 1.422 [1.143-1.768] | 0.002* |

HR, Hazard ratio; CI, confidence interval; Gal-1, galectin-1; *Statistically significant ($p$<0.05)

## Supplementary Figures

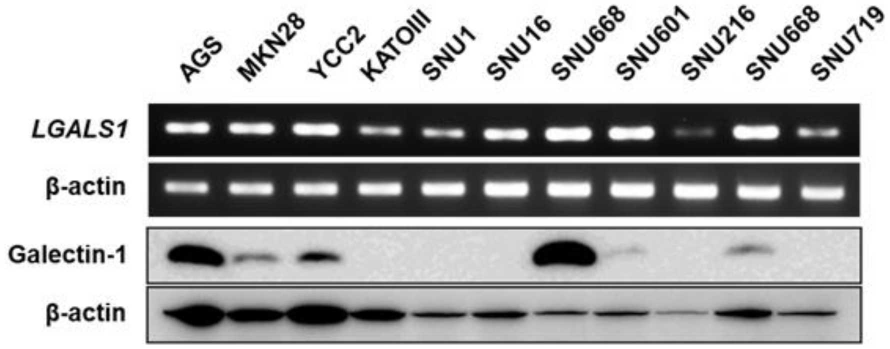


**Supplementary Figure S1. Detection of galectin-1 expression in gastric cancer cell lines.** mRNA and protein expression levels of galectin-1 were examined in 11 gastric cancer cell lines by RT-PCR and western blotting.

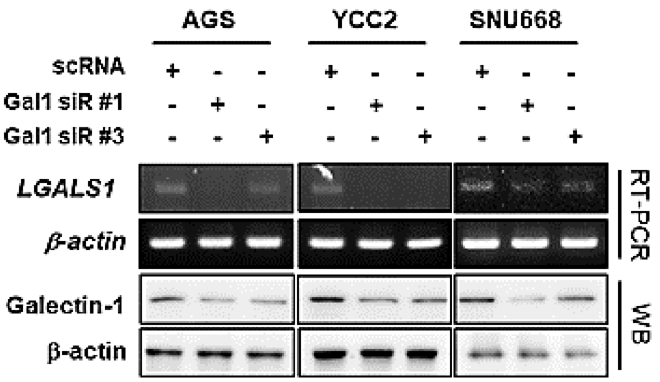


**Supplementary Figure S2. Suppression of galectin-1 expression by siRNAs in gastric cancer cell lines.** AGS, YCC2, and SNU-668 cells were transfected with scrambled control (scRNA) or two different galectin-1 siRNAs (Gal1 siR#1 and Gal1 siR#3). Cells were harvested 48 h post-transfection, and galectin-1 mRNA and protein levels were analyzed by RT-PCR and western blotting.

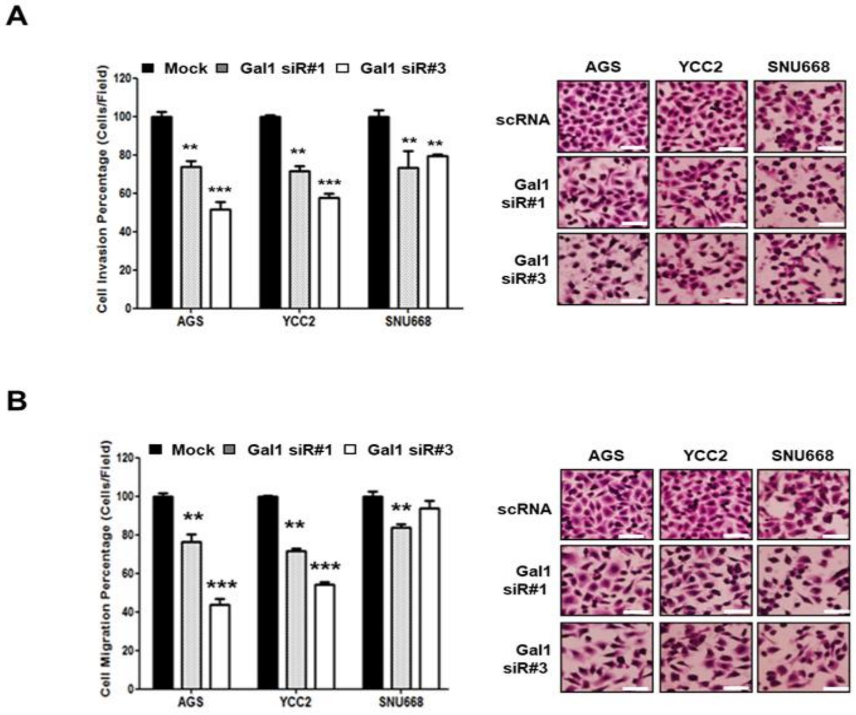


**Supplementary Figure S3. Effect of galectin-1 silencing on migration and invasion of gastric cancer cell lines.** Transwell assays were performed to evaluate (A) invasion and (B) migration in AGS, YCC-2, and SNU-668 cells transfected with scrambled control (scRNA) or galectin-1 siRNAs. Representative images (right) and quantification (left) are shown. In each group, five random fields were imaged and counted. Scale bar, 5 μm. p-values were calculated using Student's t-test (*$p < 0.05$; **$p < 0.01$; ***$p < 0.001$).

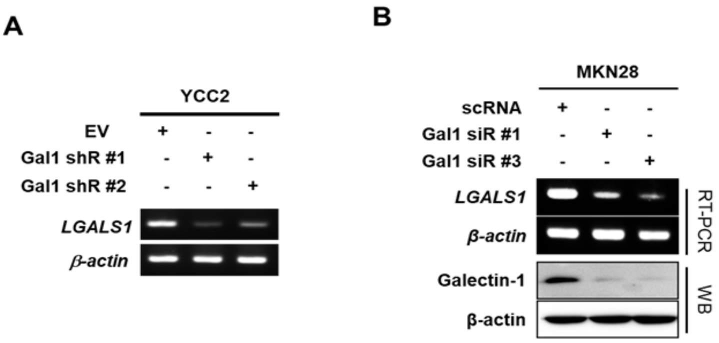


**Supplementary Figure S4. Suppression of galectin-1 expression by shRNA or siRNA in gastric cancer cell lines.** (A) YCC-2 cells were transfected with empty vector (EV) or two independent galectin-1 shRNAs. mRNA expression levels of galectin-1 were measured by RT-PCR after 48 h.

(B) MKN28 cells were transfected with scrambled control (scRNA) or two independent galectin-1 siRNAs. Galectin-1 mRNA and protein levels were assessed by RT-PCR and western blotting after 48 h.

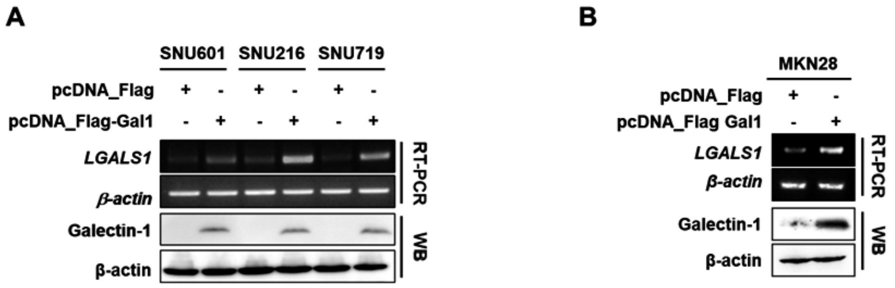


**Supplementary Figure S5. Overexpression of galectin-1 in gastric cancer cells.** (A) SNU601, SNU216, and SNU719 cells were transfected with empty vector (pcDNA_Flag) or galectin-1 overexpression vector (pcDNA_Flag-Gal1). mRNA and protein levels of galectin-1 were measured by RT-PCR and western blotting after 48 h. (B) MKN28 cells were transfected with empty vector (pLECE3) or galectin-1 overexpression vector (pLECE3-Gal1). Galectin-1 expression was confirmed by RT-PCR and western blotting after 48 h.

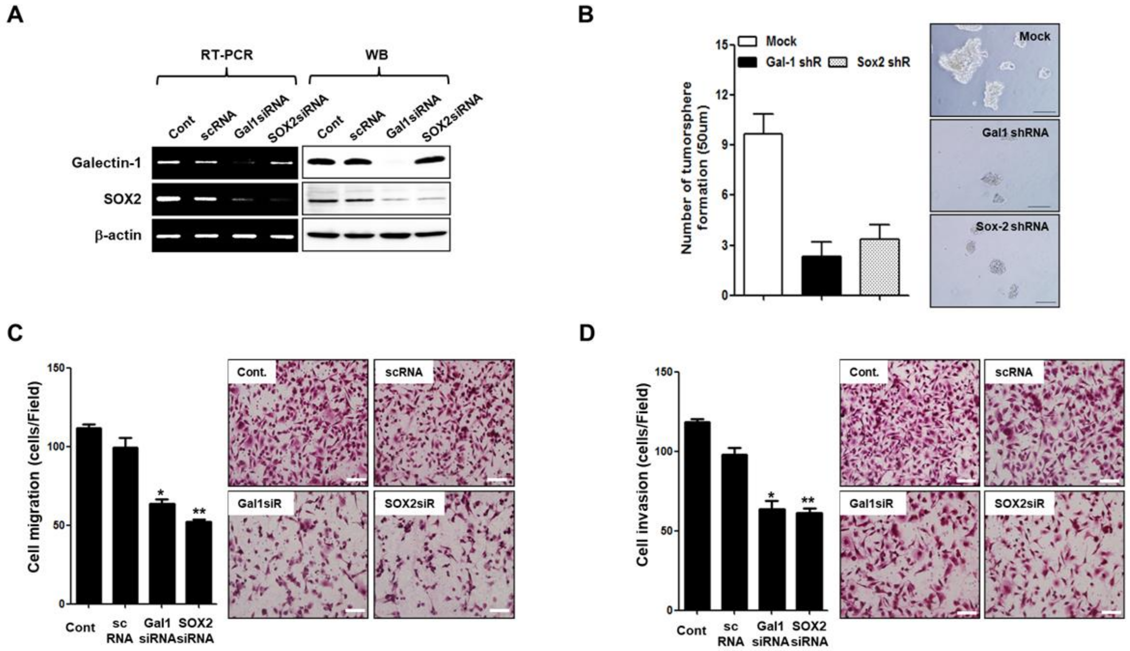


**Supplementary Figure S6. Effects of galectin-1 and SOX2 knockdown on stemness and motility in AGS gastric cancer cells.** (A) RT-PCR and western blot analysis of galectin-1 and SOX2 expression in AGS cells transfected with scRNA, galectin-1 siRNA, or SOX2 siRNA for 48 h.

(B) Tumorsphere formation assay of AGS cells transfected with galectin-1 or SOX2 siRNA. Representative images (left) and quantification of sphere number (right) are shown (magnification, ×100; mean ± SD, n = 3). Scale bar = 50 µm. (C–D) Transwell migration (C) and invasion (D) assays of AGS cells transfected with galectin-1 or SOX2 siRNA. Representative images (right) and quantification (left) are shown. Scale bar = 50 µm. Statistical significance was determined using Student's t-test (*p < 0.05; **p < 0.01; ***p < 0.001).

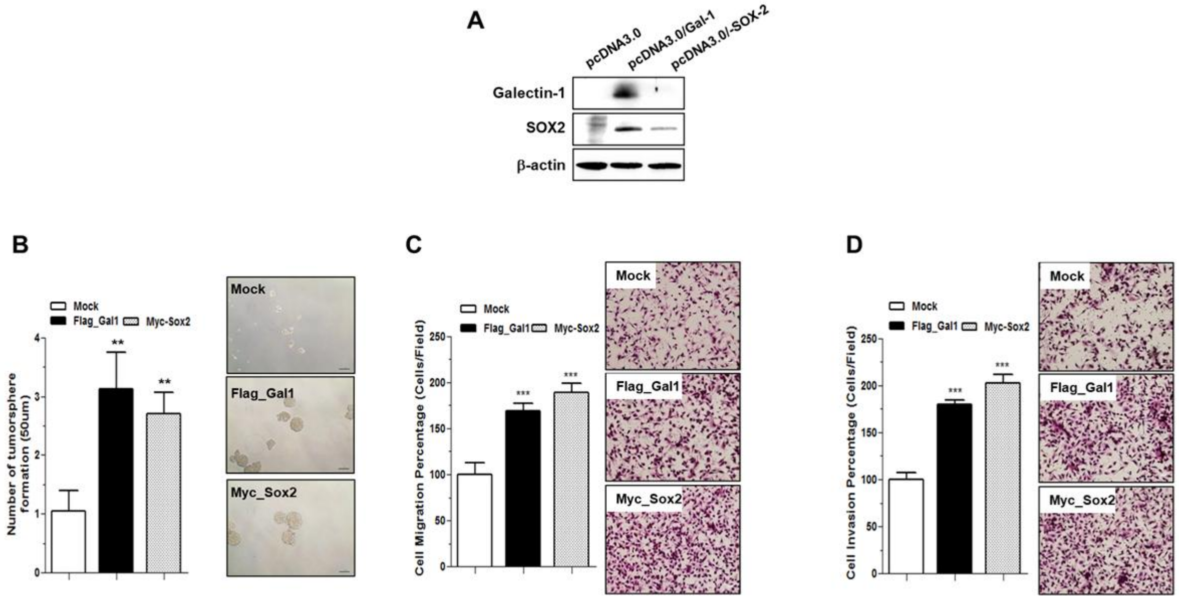


**Supplementary Figure S7. Effects of galectin-1 and SOX2 overexpression on stemness and motility in AGS gastric cancer cells.** (A) Western blot analysis of galectin-1 and SOX2 protein expression in AGS cells transfected with empty vector, pcDNA3.0-Flag-Galectin-1, or pcDNA3.0-Myc-SOX2. (B) Tumorsphere formation assay of AGS cells following overexpression of galectin-1 or SOX2. Representative images (left) and quantification of sphere number (right) are shown (magnification, ×100; mean ± SD, n = 3). Scale bar = 50 μm. (C–D) Transwell migration (C) and invasion (D) assays of AGS cells overexpressing galectin-1 or SOX2. Representative images (right) and quantification (left) are shown. Scale bar = 50 μm. Statistical significance was determined using Student's t-test (*p < 0.05; **p < 0.01; ***p < 0.001).

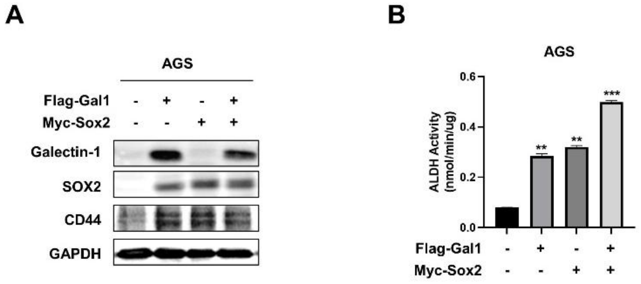


**Supplementary Figure S8. Effects of galectin-1 and SOX2 overexpression on stemness in AGS gastric cancer cells. (A**) Western blot analysis of galectin-1, SOX2 and CD44 protein expression in AGS cells transfected with empty vector, pcDNA3.0-Flag-Galectin-1, or pcDNA3.0-Myc-SOX2. (B) Quantification of ALDH activity in AGS cells overexpressing vector, pcDNA3.0-Flag-Galectin-1, or pcDNA3.0-Myc-SOX2. Statistical significance was determined using Student's t-test (**p < 0.01; ***p < 0.001).

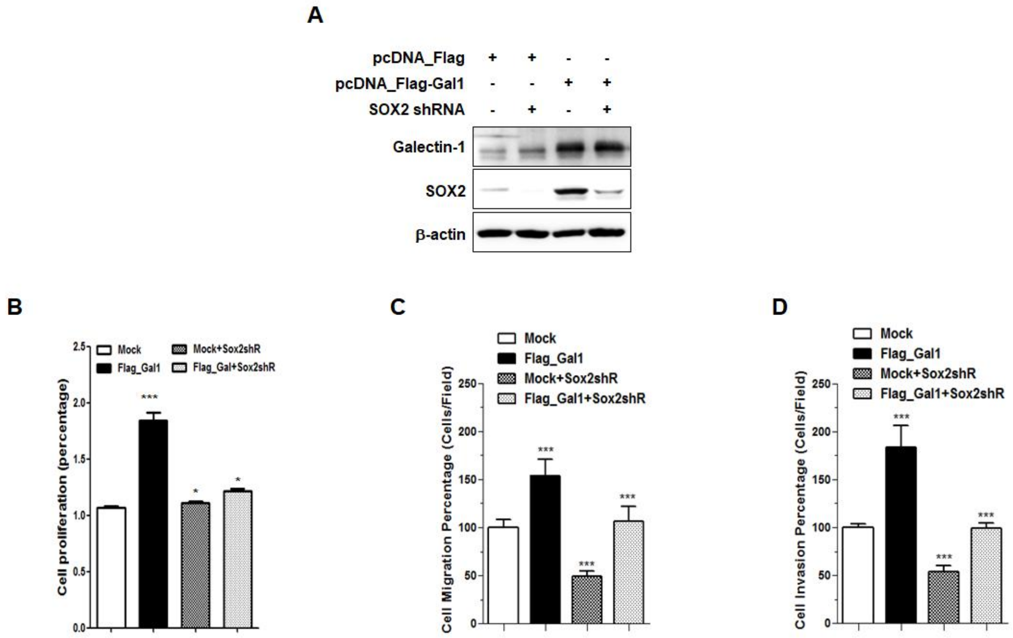


**Supplementary Figure S9. SOX2 silencing abrogates the proliferative and motility effects induced by galectin-1 overexpression in AGS gastric cancer cells.** (A) Western blot analysis of SOX2 and galectin-1 expression in AGS cells co-transfected with empty vector, galectin-1 overexpression vector, and/or SOX2 shRNA. (B) Cell proliferation was assessed using the WST assay in the indicated AGS cell groups. Data are presented as mean ± SD (n = 3). (C–D) Transwell migration (C) and invasion (D) assays were performed in AGS cells co-transfected with galectin-1 overexpression vector and SOX2 shRNA. Representative images and quantification are shown. Scale bar = 50 μm. Statistical significance was determined using Student's t-test (*p < 0.05; **p < 0.01; ***p < 0.001).

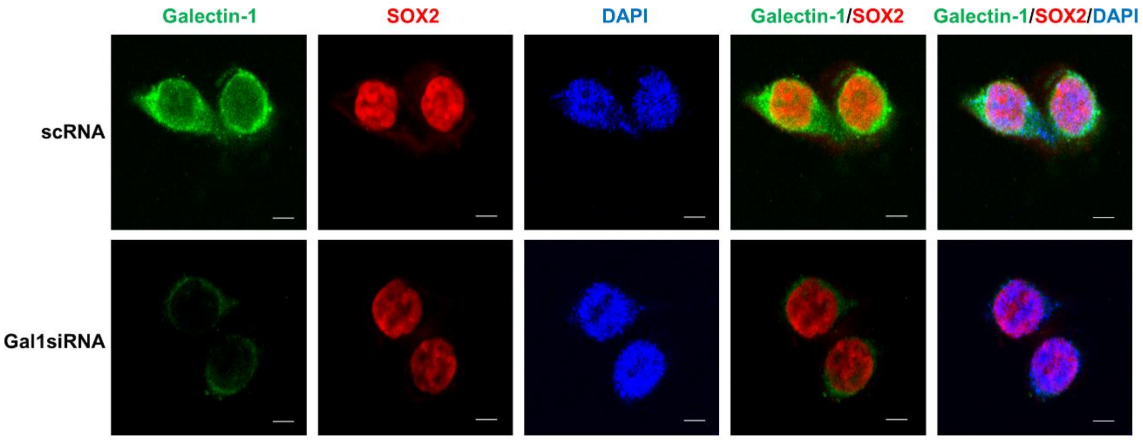


**Supplementary Figure S10. Nuclear co-localization of galectin-1 and SOX2 in gastric cancer cells.** AGS cells were transfected with scRNA or galectin-1 siRNA #1. AGS cells were transfected with scRNA or galectin-1 siRNA #1. IF for Galectin-1 (green) and SOX2 (red) was performed and visualized using confocal microscopy. Nuclei were stained with 4′,6-diamidino-2-phenylindole (blue). Galectin-1/SOX2, merged image of Galectin-1 and SOX2; Galectin-1/SOX2/DAPI, merged image of Galectin-1, SOX2, and DAPI. Image magnification, × 400; scale bar, 20 μm.

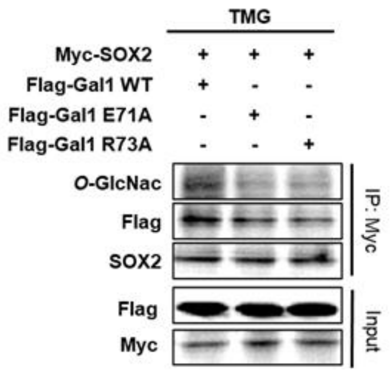


**Supplementary Figure S11. Analysis of SOX2 O-GlcNAcylation under wild-type or mutant galectin-1 expression.** Gastric cancer cells were transfected with wild-type galectin-1 or mutants (E71A or R73A). Cell lysates were subjected to immunoprecipitation using an anti-Myc antibody, followed by immunoblotting with an anti–O-GlcNAc antibody to assess O-GlcNAcylation levels of SOX2.

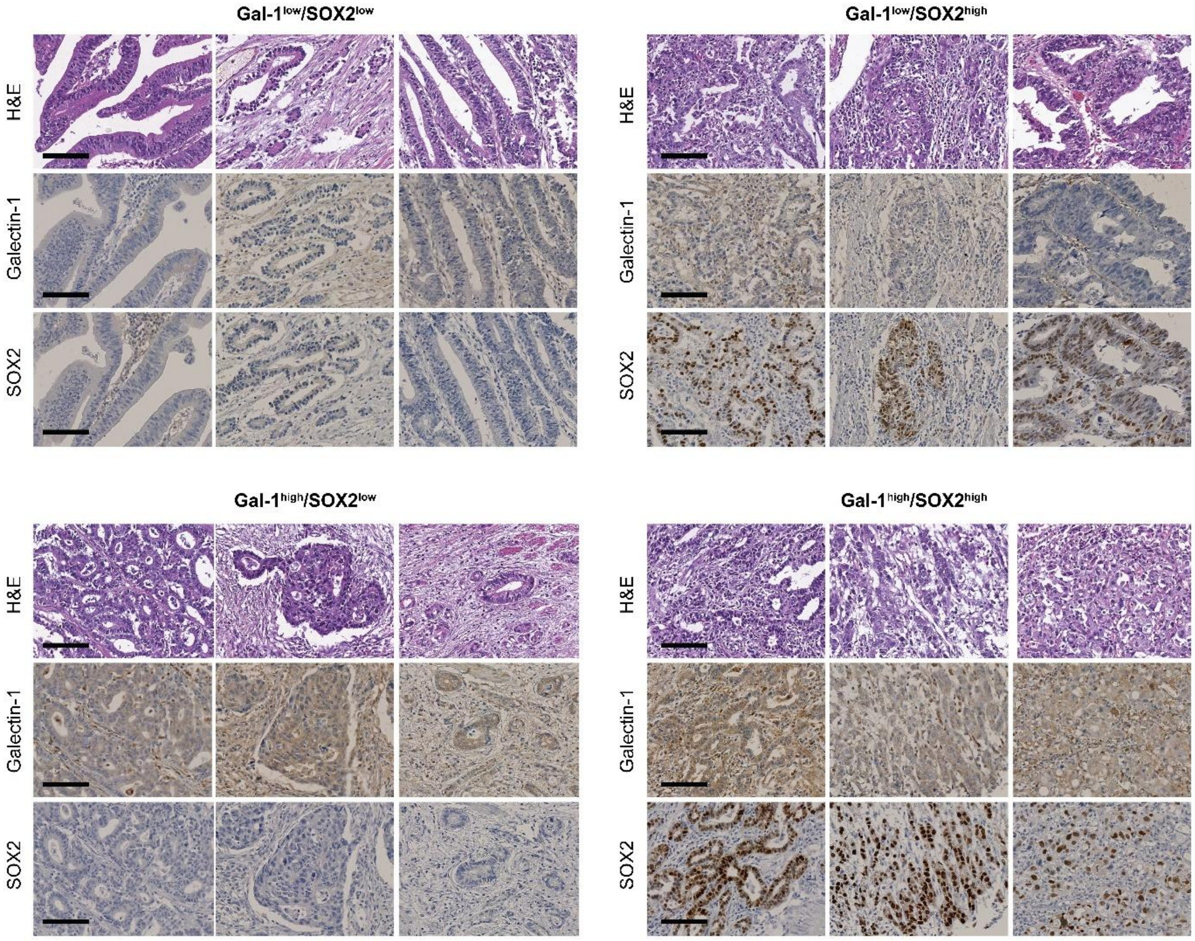


**Supplementary Figure S12.** Representative hematoxylin and eosin (H&E) staining and immunohistochemical detection of Galectin-1 and SOX2 illustrating four distinct expression patterns. Three formalin-fixed, paraffin-embedded gastric cancer cases per group are shown at high magnification. Scale bar, 100 $\mu$m.
